# Interfacial Enrichment and Demetallation of a Zinc Porphyrin in the Ionic Liquid [C_4_C_1_Im][PF_6_]

**DOI:** 10.1002/cphc.70318

**Published:** 2026-04-20

**Authors:** Alisson Ceccatto, Federico J. Williams, Florian Maier, Hans‐Peter Steinrück

**Affiliations:** ^1^ Lehrstuhl für Physikalische Chemie 2 Friedrich‐Alexander‐Universität Erlangen‐Nürnberg Erlangen Germany; ^2^ Departamento de Química Inorgánica Analítica y Química Física Facultad de Ciencias Exactas y Naturales Universidad de Buenos Aires Buenos Aires Argentina; ^3^ Instituto de Química Física de los Materiales Medio Ambiente y Energía CONICET‐Universidad de Buenos Aires Buenos Aires Argentina

## Abstract

Understanding and controlling the surface composition of metalloporphyrins in ionic liquids (ILs) is crucial for designing photoactive materials. Here, we investigate the interfacial behavior of zinc‐didodecylporphyrin molecules (Zn‐DDP) dissolved in the hydrophobic ionic liquid 1‐butyl‐3‐methylimidazolium hexafluorophosphate [C_4_C_1_Im][PF_6_] using angle‐resolved X‐ray photoelectron spectroscopy (ARXPS). Upon mild heating, Zn‐DDP undergoes spontaneous demetallation, evidenced by the appearance of aminic (–NH–) and iminic (=N–) nitrogen XPS signals characteristic of the free‐base porphyrins. Partial hydrolysis of [PF_6_]^‐^ anions produces phosphate species that contribute to the demetallation process. Concentration‐dependent measurements reveal extremely high surface enrichment of porphyrins at the IL/vacuum interface, reaching surface saturation already at about 0.50%_mol_ Zn‐DDP content in the bulk. The dodecyl chains of porphyrins at the outer surface are oriented toward the vacuum in a buoy‐like configuration. Temperature‐dependent ARXP spectra reveal that for unsaturated interfaces, surface enrichment increases with increasing temperature. Our findings provide molecular‐level insights to guide the design of porphyrin‐based photoactive interfaces in ionic liquid systems.

## Graphical abstract

Upon dissolution of zinc porphyrins with long dodecyl chains in the ionic liquid [C_4_C_1_Im][PF_6_], partial [PF_6_]^‐^ hydrolysis occurs along spontaneous porphyrin demetallation. Driven by their buoy‐like chains, the free‐base porphyrins extremely enrich at the outermost surface as proven by angle‐resolved X‐ray photoelectron spectroscopy, an interesting starting point for designing porphyrin‐based photoactive interfaces in ionic liquids.

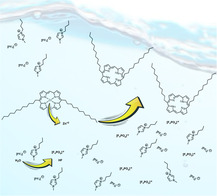

## 1 Introduction

Ionic liquids (ILs) have emerged as versatile media for catalysis and photochemical processes due to their unique physicochemical properties such as thermal stability, molecular tunability, and extremely low vapor pressure [1, 2, 3, 4, 5, 6]. The properties are mainly governed by the different Coulombic, van der Waals, and *π–* *π* interactions present in the ILs [7]. This leads to different solvation and stabilization effects on solutes, leading to distinct local environments [8, 9]. Notably, the interfacial behavior of ILs is a key factor in bi‐phasic catalysis and can also be explored in photochemical processes [10, 11, 12]. Recently, imidazolium‐based ILs were used to tune the light response of photochromic diarylethenes thin films by increasing the ionic conductivity of the synthesized material [13], showing that—in addition to heterogeneous catalysis—ILs are suitable media to design and tune light‐responsive materials [14, 15, 16].

In the context of light‐driven processes, porphyrin‐based molecular systems occupy a central position. Porphyrins are organic macrocycles that play key roles in biological processes and exhibit intense absorption in the visible and ultraviolet regions due to their extended conjugated *π*‐system [17, 18, 19]. This strong light‐harvesting ability, combined with the chemical versatility provided by diverse substituents, opens broad possibilities for designing functional materials. Their coordination with transition metals such as Pd, Mn, Co, or Ni further tunes their redox and photochemical properties [20, 21, 22]. Over the past decades, numerous studies have explored porphyrins in photoactive materials for photocatalysis [23], dye‐sensitized solar cells [24, 25, 26], and optoelectronics [27]. However, their performance in conventional organic solvents is often limited by photodegradation and thermal instability [28]. Ionic liquids (ILs) offer an appealing alternative owing to their advantageous physicochemical properties, making them suitable media for applications such as dye‐sensitized solar cells [29]. Furthermore, the use of ILs can reduce molecular aggregation through ionic screening and modulate electron‐transfer dynamics via their heterogeneous polarity [30]. Despite this potential, the interfacial behavior and mechanistic aspects of porphyrins in ILs are still insufficiently investigated.

Recently, metal–organic complexes have been investigated in ILs within the framework of supported ionic liquid phase (SILP) catalysis [31]. In these systems, the catalyst consists of a metal complex dissolved in a thin IL film. The IL/gas or IL/vacuum interface plays a critical role in determining catalytic performance and efficiency. By introducing functional groups into the metal complexes, their interfacial behavior can be deliberately tuned. For instance, the incorporation of perfluorinated substituents in Pt complexes promotes migration toward the IL/vacuum interface, enhancing their local concentration and, potentially, their catalytic activity [32]. A similar effect has been reported for Ru complexes bearing long alkyl chains dissolved in [C_2_C_1_Im][OAc] and [C_4_C_1_Im][PF_6_], where pronounced surface enrichment arises from the amphiphilic character of the chains [33, 34]. Thus, in ILs, metal complexes with suitable functional substituents can undergo surface segregation, commonly referred to as the “buoy effect” [35, 36]. This phenomenon significantly alters the molecular concentration near the interface and may be extended to photoinduced processes, as many light‐driven reactions occur at or close to interfaces [23, 37, 38]. Applying this still largely unexplored buoy concept to suitable porphyrin systems dissolved in ILs could thus open new avenues for designing advanced photochemical architectures. It is also important to note from an analytical point of view that strongly enriched reactive species in ILs also offer the unique possibility to monitor in‐situ chemical reactions in great detail by means of XPS under well‐defined UHV conditions, even when the bulk concentration of these species is very low.

**FIGURE 1:**
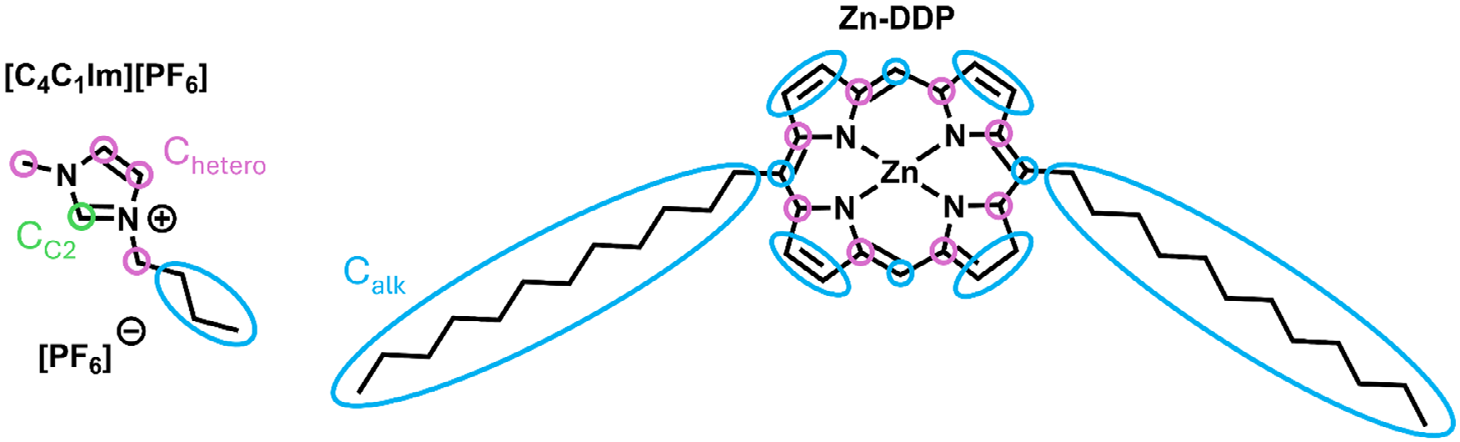
Molecular structures of the [C_4_C_1_Im][PF_6_] IL and the Zn‐DDP porphyrin, highlighting the different C species.

In this work, we investigate the interfacial behavior of zinc‐didodecylporphyrin (Zn‐DDP) in the hydrophobic ionic liquid 1‐butyl‐3‐methylimidazolium hexafluorophosphate [C_4_C_1_Im][PF_6_] (Figure 1). The molecule contains a Zn^2+^ center coordinated within the tetrapyrrole ring that bears two C_12_ alkyl chains attached at the meso positions (Figure 1). To increase the solubility of the porphyrins in the IL, we heated the solutions to 423–453 K in the presence of tetrahydrofuran, leading to a complete dissolution of the porphyrins in [C_4_C_1_Im][PF_6_]. Angle‐resolved X‐ray photoelectron spectroscopy (ARXPS) was employed to quantify the interfacial composition as a function of temperature and Zn‐DDP concentration. Our findings provide direct insights into the interfacial behavior and chemical reactivity of porphyrins in ionic liquids as revealed by ARXPS, offering new opportunities for designing photochemically active IL‐based interfaces.

## 2 Results and Discussion

**FIGURE 2:**
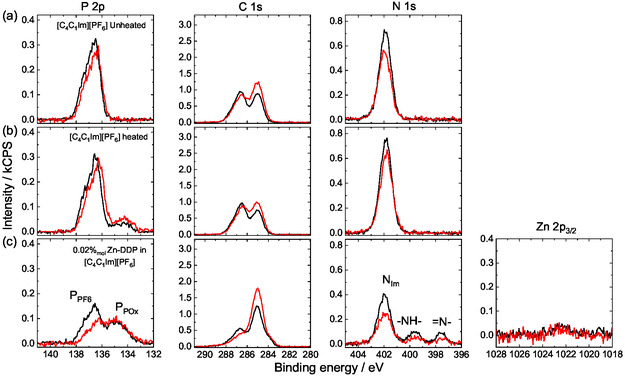
Angle‐resolved XP spectra for the C 1s, N1s, P 2p, and Zn 2p_3/2_ regions for the neat [C_4_C_1_Im][PF_6_] IL (a) unheated and (b) heated to 398 K for ~5 h and for the (c) prepared solution (including heating to 443 K for ~5 h) with a Zn‐DDP concentration of 0.02%_mol_ in [C_4_C_1_Im][PF_6_] measured in 0° (black, more bulk‐sensitive) and in 80° (red, more surface‐sensitive).

Figure 2a shows the ARXP spectra of the P 2p, C 1s, and N 1s regions for the unheated neat [C_4_C_1_Im][PF_6_] measured at 0° (black, more bulk‐sensitive; for more details, see Experimental) and 80° (red, more surface‐sensitive) emission angles (for the full set of spectra of all relevant regions and detailed peak assignments, see Figure S1a,b of Supporting Information (SI), respectively). All regions display the expected features: the spin–orbit‐split P 2p peak (with the 2p_3/2_ component at 136.5 eV binding energy) corresponding to [PF_6_]^‐^ anions, the N 1s peak from the [C_4_C_1_Im]^+^ cation at 402.0 eV, and the corresponding C 1s peaks between 285.0 and 287.0 eV, with a slight enrichment of aliphatic carbon component at 285.0 eV at 80°, as was reported earlier [39]. Upon heating [C_4_C_1_Im][PF_6_] at 398 K for several hours under ambient conditions, the XP spectra reveal a marked change. As shown in Figure 2b, the P 2p region exhibits two distinct components: a dominant high‐binding‐energy feature assigned to [PF_6_]^‐^ anions at 136.5 eV and a minor component shifted toward lower binding energy at 134.2 eV. The latter component is attributed to phosphate (PO_*x*_) species. XPS measurements of the O 1s region shown in Figure S2 of the SI confirm the presence of oxygen with a P:O ratio of approximately 1:3. The formation of phosphate species upon heating likely arises from partial hydrolysis of [PF_6_]^‐^ due to trace amounts of water in the IL; note that the neat IL (and also the solutions discussed below) were heated under ambient conditions. This interpretation is consistent with previous reports showing that hydrolysis of [PF_6_]^‐^ occurs above 343 K, leading to the formation of PO_*x*_ species [40]. The P 2p peak shows a moderate core‐level shift between the measurements at 0° and 80° emission angles due to their chemical environments (about −0.2±0.2 eV in 80°). This behavior is mainly attributed to the different chemical environments of the P species at the outer surface as compared to the bulk, as reported previously [41].

A key experimental challenge lies in achieving molecular solubility of Zn‐DDP to obtain homogeneous solutions. Zn‐DDP exhibits very low solubility in [C_4_C_1_Im][PF_6_] due to its weak polarity. Indeed, no apparent porphyrin dissolution in the neat IL could visually be detected (which would lead to color changes even at very low concentrations; see Figure S3–S9 in the supporting information) even after days of intense stirring under ambient conditions, and the XPS signals of the resulting liquid containing dispersed porphyrin particles (see Figure S10) were identical to the ones of the neat IL. Therefore, obtaining solutions with appreciable Zn‐DDP concentrations required the use of tetrahydrofuran (THF) as a co‐solvent, followed by stirring for ∼5 h at 423–453 K under ambient conditions with concomitant THF evaporation. This procedure yields homogeneous Zn‐DDP solutions in [C_4_C_1_Im][PF_6_] with concentrations controlled in the range of 0.02–0.50%_mol_. Figure 2c shows XP spectra measured at 0° (black) and 80° (red) emission angles for a 0.02%_mol_ Zn‐DDP solution in [C_4_C_1_Im][PF_6_]. The N 1s core level shows three nitrogen components. As in the neat (Figure 2a) and heated (Figure 2b) IL, a main peak appears at 402.0 eV, which is assigned to the imidazolium nitrogen of the [C_4_C_1_Im]^+^ cation. Two additional peaks are observed at 399.7 and 397.6 eV with a 1:1 intensity ratio. To assign these low‐binding‐energy components, we note that the N 1s spectrum of free‐base tetraphenylporphyrins exhibits two peaks at 400.1 and 398.0 eV in a 1:1 ratio, corresponding to aminic (–NH–; also often denoted also pyrrolic) and iminic (=N–) nitrogen, respectively [42]. By analogy, the features at 399.7 and 397.6 eV are attributed to –NH– and =N– originating from dissolved free‐base porphyrins, which indicates that the Zn‐DDP molecules had been demetallated. This assignment is further supported by the Zn 2p_3/2_ XP spectrum in Figure 2c, which shows only a very weak Zn signal with a Zn:N_–NH–_ ratio measured in 0° of approximately 1:10, corresponding to a five‐fold lower Zn concentration relative to the demetallated porphyrins. The C 1s spectrum of the Zn‐DDP solutions displays an intense peak at 285.0 eV, predominantly arising from the C_12_ alkyl chains of the porphyrins. This peak increases in intensity at 80° emission (surface‐sensitive) compared to 0° (bulk‐sensitive), indicating surface enrichment of the porphyrins at the IL/vacuum interface in a buoy‐like fashion. This effect is even more evident given that N species from the porphyrin macrocycle are detected at a nominal concentration as low as 0.02%_mol_. At this concentration, the IL:porphyrin ratio is ∼5000, whereas the XPS N_Im_:N_NH_ ratio is ∼4.0, corresponding to an enrichment by about three orders of magnitude for porphyrin molecules relative to imidazolium cations at the IL/vacuum interface.

The XPS data discussed above clearly show that Zn‐DDP molecules lose their Zn^2+^ metal center in [C_4_C_1_Im][PF_6_] solutions and segregate to the IL/vacuum interface. Surface enrichment is driven by the C_12_ alkyl chains, which lower the surface tension of the solution and therefore migrate to the interface. Typically, in methanolic solutions, Zn‐porphyrin demetallation requires acidic conditions (pH < 4) [43]. In the present case, however, demetallation must be rationalized considering that to dissolve Zn‐DDP, solutions were heated under ambient conditions, where the IL contains residual water. This leads to hydrolysis of [PF_6_]^‐^, as evidenced by the phosphate‐like species observed in the P 2p XP spectrum and consistent with previous studies [40], producing HF and F_*y*_PO_*x*_^‐z^ (*z* = 5‐2x‐y) anions. Although HF can remain bound to ILs and provide acidic conditions for demetallation [44], it should evaporate under the heating conditions employed. We therefore associate Zn‐DDP demetallation with the interaction between the Zn^2+^ center in the porphyrin molecules and the phosphate species formed during heating, along with protonation of the macrocycle center. The majority of Zn^2+^ species in solution are likely present as Zn‐phosphate and/or Zn‐fluoride complexes (note that the concentration of such Zn‐species is well below XPS detection as long as they do not enrich at the surface). This proposal is consistent with recent studies showing that phosphate species inhibit metallation of porphyrins with Cu^2+^ ions [45].

**FIGURE 3:**
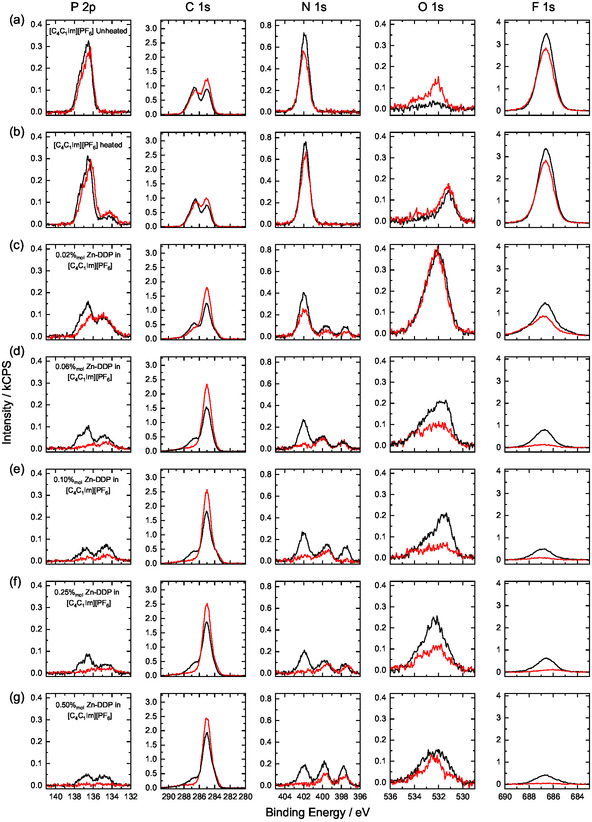
ARXP spectra (0° black, 80° red) for P 2p, C 1s, N 1s, O 1s, and F 1s regions of the neat [C_4_C_1_Im][PF_6_] IL (a) unheated and (b) after heating at 398 K and for solutions of a Zn‐DDP concentration of (c) 0.02%_mol_, (d) 0.06%_mol_, (e) 0.10%_mol_, (f) 0.25%_mol_, and (g) 0.50%_mol_ in [C_4_C_1_Im][PF_6_]. The oxygen signal in (a) is due to small silicone contamination present at the surface [1].

To gain further insight into these mechanisms, we conducted a systematic investigation as a function of Zn‐DDP concentration, varying from 0.02%_mol_ to 0.50%_mol_. All solutions were prepared following the same procedure described above. Figure 3 displays the XP spectra of the C 1s, N 1s, O 1s, F 1s, and P 2p regions for all Zn‐DDP solutions, together with the spectrum of the nonheated and the heated [C_4_C_1_Im][PF_6_] IL. Complete XP spectra for all relevant regions are provided in Figures S1–S9. For comparison, Figure 3a shows the data for the neat, unheated [C_4_C_1_Im][PF_6_] IL. For all Zn‐DDP solutions, the XP spectra are dominated by a C 1s peak at 285.0 eV, due mainly to the C_12_ side chains in the macrocycle. In every case, the spectra recorded at 80° emission (surface‐sensitive, shown in red) display a more intense signal than those recorded at 0° emission (bulk‐sensitive, shown in black), indicating that the DDP molecules are enriched at the IL/vacuum interface across the entire concentration range investigated, adopting a buoy‐like configuration. This conclusion is further supported by the pronounced attenuation of IL‐specific signals in the N 1s, F 1s, and P 2p regions compared with the neat [C_4_C_1_Im][PF_6_] sample. As noted above, the N 1s spectra exhibit two peaks corresponding to the iminic (=N–) and aminic (–NH–) nitrogen atoms of the porphyrin, indicating that demetallation occurs in all cases. Furthermore, the 0° emission spectra (black) show slightly more intense iminic and aminic peaks than the 80° spectra (red), suggesting that the porphyrins adopt an orientation in which the C_12_ chains point toward the IL/vacuum interface, with the metal‐free macrocycle positioned below (see Figure S11 and Table S2). This preferred orientation is consistent with recent studies on the surface enrichment of metal complexes functionalized with long alkyl chains in ILs, where comparable molecular arrangements were observed [33, 34]. It is important to note that no Zn species were detected in any of the solutions examined, with the exception of the 0.02%_mol_ sample, where a small Zn‐related peak is observed as discussed above (Figures S3–S9 in the SI). Finally, note that the P 2p_3/2_ peak at 134.2 eV and the O 1s peak at ∼532.0 eV in the spectra shown in Figure 3 indicate the presence of F_*y*_PO_*x*_^‐z^ species in all solutions, arising from the hydrolysis of PF_6_^‐^ anions during the heating step required to dissolve Zn‐DDP. The neat [C_4_C_1_Im][PF_6_] IL has a nominal N_Im_:P ratio of 2:1. However, in the presence of phosphate species, the experimental N_Im_:P ratio matches the nominal value only when both PF_6_^‐^ and PO_*x*_ species are considered, as shown in Table 1, confirming that PF_6_^‐^ hydrolysis generates phosphate anions.

**TABLE 1:** Ratio of the N_Im_:P_total_ considering the contribution from both P species (spectra measured in 0°).

**Sample**	**N_Im_:P_total_, 0°**
0.02%_mol_	1.9
0.06%_mol_	2.0
0.10%_mol_	2.0
0.25%_mol_	2.0
0.50%_mol_	2.8
[C_4_C_1_Im][PF_6_] – 398 K	2.8

**FIGURE 4:**
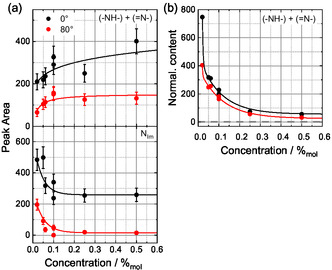
(a) Peak area for the sum of −NH− and =N− (top) and N_Im_ (bottom) species obtained from the N 1s signal in 0° (black) and 80° (red) and (b) normalized content for the sum of −NH− and =N− species for concentrations ranging from 0.02%_mol_ to 0.50%_mol_.

Figure 4a shows the peak areas for the sum of the porphyrin =N– and –NH– signals (top) and the IL N_Im_ signal (bottom) at both emission angles (0° and 80°) as a function of Zn‐DDP concentration (lines are guides to the eye). As expected, the peak area associated with the porphyrin molecules increases, whereas that of the IL‐related signal decreases with increasing Zn‐DDP concentration. Already at 0.1%_mol_, approximately 90% of the saturation value is reached, indicating that the IL/vacuum interface becomes saturated at relatively low Zn‐DDP concentrations. The N_Im_ signal also reaches saturation at similar concentrations, suggesting that IL ions at the interface are progressively replaced by porphyrin molecules. A similar behavior is observed for the F_*y*_PO_*x*_^‐z^ species (not shown), which decreases in the same manner as the N_Im_. Figure 4b displays the summarized =N– and –NH– content normalized to the nominal bulk concentration (Table S1), representing the ratio of the experimental and nominal porphyrin contents at 0° (black) and 80° (red) as a function of Zn‐DDP concentration. A value of 1 (indicated by the dashed line in Figure 4b) corresponds to homogeneously distributed and randomly oriented DDP molecules. For both bulk‐sensitive (0°) and surface‐sensitive (80°) measurements, the normalized content for the sum of –NH– and =N− increases substantially as the Zn‐DDP concentration decreases. At 0°, the enrichment factor is ∼750 for the 0.02%_mol_ solution and decreases to ∼55 for the 0.50%_mol_ solution. In all cases, the normalized (–NH– + =N−) content is higher at 0° than at 80°, confirming that the porphyrin macrocycle is located beneath the C_12_ alkyl chains.

**FIGURE 5:**
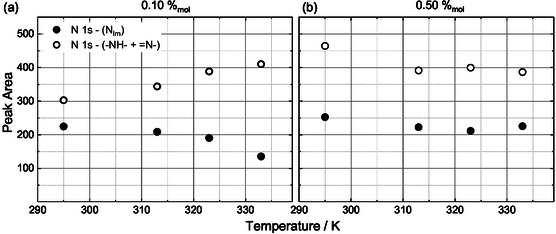
Temperature dependence of the normalized peak areas for the sum of −NH− and =N− (open symbols), and of N_Im_ (closed symbols) species obtained from the N 1s signals recorded at 0° for solutions with a Zn‐DDP concentration of (a) 0.10%_mol_ and (b) 0.50%_mol_.

Finally, we investigated the effect of temperature on surface enrichment. Figure 5 shows the temperature dependence (295–343 K) of the porphyrin nitrogen (sum of −NH− and =N−) and N_Im_ signals for the bulk‐sensitive (0°) measurements of the 0.10%_mol_ and 0.50%_mol_ solutions. The corresponding surface‐sensitive (80°) data are provided in Figure S11, where the –NH– and =N– signals in Figure 5a remains constant, and the N_Im_ signal lies below the detection limit. Interestingly, for the 0.10%_mol_ solution, the porphyrin nitrogen signals irreversibly increase in 0° ∼30%, while the N_Im_ signal decreases by ∼50%. Because no changes are observed in the 80° spectra, we attribute this behavior to diffusion of porphyrin molecules toward the IL/vacuum interface as temperature increases, until surface saturation is reached. As the concentration of porphyrins at the interface rises, the IL‐specific signals decrease due to the replacement of [C_4_C_1_Im]^+^ cations by the porphyrin molecules. In contrast, the 0.50%_mol_ solution shows no measurable temperature dependence over the same range, with both −NH−, =N− and N_Im_ signals remaining essentially constant. This observation is consistent with the surface being already saturated with porphyrins at this concentration. These results demonstrate that temperature can be used to modulate surface enrichment and tune the interfacial composition of unsaturated systems.

## 3 Conclusions

X‐ray photoelectron spectroscopy measurements reveal that Zn‐DDP porphyrins undergo spontaneous demetallation when dissolved in [C_4_C_1_Im][PF_6_]. This is evidenced by the absence of Zn signals and the presence of N species associated with the aminic and iminic nitrogens of free‐base porphyrins. The demetallation reaction is attributed to the presence of phosphate species in the IL, which coordinate to the Zn^2+^ center of the porphyrin macrocycle. These phosphate species form through hydrolysis of PF_6_^‐^ anions during the heating step required for solution preparation in the presence of residual water. Furthermore, ARXPS measurements demonstrate pronounced surface enrichment of the metal‐free porphyrin molecules at the IL/vacuum interface, adopting a buoy‐like orientation in which the aliphatic C_12_ chains point toward the surface, while the porphyrin macrocycle resides closer to the bulk. The extent of surface segregation is strongly concentration‐dependent: Enrichment factors reach ∼750 at 0.02%_mol_, with interfacial saturation already achieved at 0.10%_mol_. For unsaturated solutions, temperature‐dependent measurements show a further increase in surface enrichment upon heating to 343 K. Together, the findings provide the first direct evidence of porphyrin surface enrichment in ionic liquids and highlight the critical role of phosphate species in driving demetallation. This understanding of interfacial mechanisms and molecular organization is essential for the rational design of photoactive materials based on porphyrins.

## 4 Experimental

### 4.1 Materials and Sample Preparation

Zinc‐didodecylporphyrin molecules (Zn‐DDP) from PorphyChem (France), 1‐Butyl‐3‐methylimidazolium hexafluorophosphate ([C_4_C_1_Im][PF_6_]) from IoLiTec (Germany, purity higher than 99% with water content below 250 ppm and halide content below 100 ppm), and all other chemicals used in this work were commercially purchased and used without further purification.

Zn‐DDP IL solutions were prepared by stirring a ∼1:1 IL:Zn‐DDP/THF mixture for 5 to 8 h while heating at 423‐453 K. The resulting solutions were then introduced in the UHV system and degassed for at least 12 h prior to measurements.

### 4.2 ARXPS Measurements and Data Evaluation

The ARXPS measurements were performed in the dual analyzer system for surface analysis (DASSA) [46]. This unique experimental setup is equipped with two analyzers mounted at 0° and 80° with respect to the surface plane. In this configuration, the XP spectra are recorded simultaneously at different information depths: In 0°, the information depth in ILs is between 7 and 9 nm depending on the photoelectrons’ kinetic energy (“bulk‐sensitive spectra”), while in 80°, only the outermost 1‐1.5 nm are probed (“surface‐sensitive spectra) [46]. The binding energy scale was referenced to the C 1s signal of aliphatic carbon at 285.0 eV, similar to previous reports [33, 34]. All XP spectra recorded at 80° were scaled up by a geometric factor to compensate for the intrinsic lower intensity compared to 0°, as discussed in more detail [46]. This procedure allows us to directly correlate the observed intensity differences with surface enrichment or depletion effects. For the quantitative analysis, the measured intensities were corrected using the appropriate atomic sensitivity factors (ASFs) [47].

## Supporting Information

Additional supporting information can be found online in the Supporting Information section.

## Funding

This work was supported by the Deutsche Forschungsgemeinschaft (Grant Project‐ID431791331 – SFB 1452), the Secretaría de Ciencia y Técnica, Universidad de Buenos Aires, the Consejo Nacional de Investigaciones Científicas y Técnicas (Grant PIP 2021), and the Alexander von Humboldt‐Stiftung (Grant Humboldt Research Fellowship for postdocs).

## Conflicts of Interest

The authors declare no conflicts of interest

## Supporting information

Supplementary Material

## Data Availability

The data that support the findings of this study are openly available in zenodo at https://10.5281/zenodo.17769558.
