# Supplementary material for: Interfacial Enrichment and Demetallation of a Zinc Porphyrin in the Ionic Liquid [C4C1Im][PF6]
Source: Chemphyschem. 2026 Apr 20;27(8):e70318. doi: 10.1002/cphc.70318 (PMC13095400; doi:10.1002/cphc.70318)
Supplement: Supplementary file 1 — Supplementary Material [file CPHC-27-e70318-s001.pdf]

Supporting Information

Interfacial Enrichment and Demetallation of a Zinc Porphyrin in the Ionic Liquid [C_4C_1Im][PF_6]

Alisson Ceccatto[a]*, Federico J. Williams[b,c], Florian Maier [a], Hans-Peter Steinrück [a]*

[a] Lehrstuhl für Physikalische Chemie 2, Friedrich-Alexander-Universität Erlangen-Nürnberg, Egerlandstr. 3, 91058 Erlangen, Germany

[b] Departamento de Química Inorgánica, Analítica y Química Física, Facultad de Ciencias Exactas y Naturales, Universidad de Buenos Aires, Buenos Aires, Argentina

[c] Instituto de Química Física de los Materiales, Medio Ambiente y Energía, CONICET-Universidad de Buenos Aires, Buenos Aires, Argentina

*Corresponding Authors: hans-peter.steinrueck@fau.de, alisson.ac.ceccatto@fau.de.

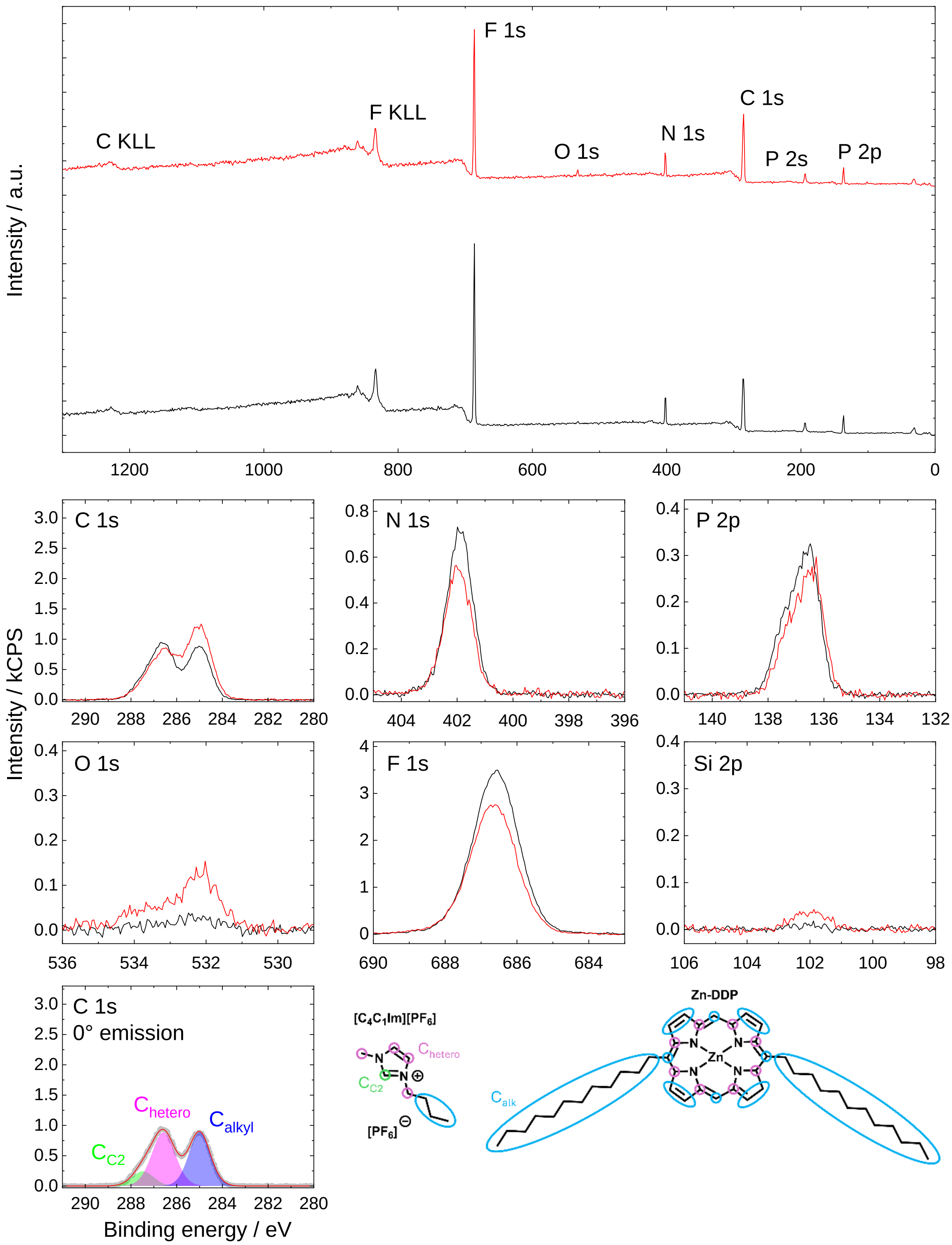

[C_4C_1Im][PF_6] Unheated

a)

$[C_4C_1Im][PF_6]$ Heated

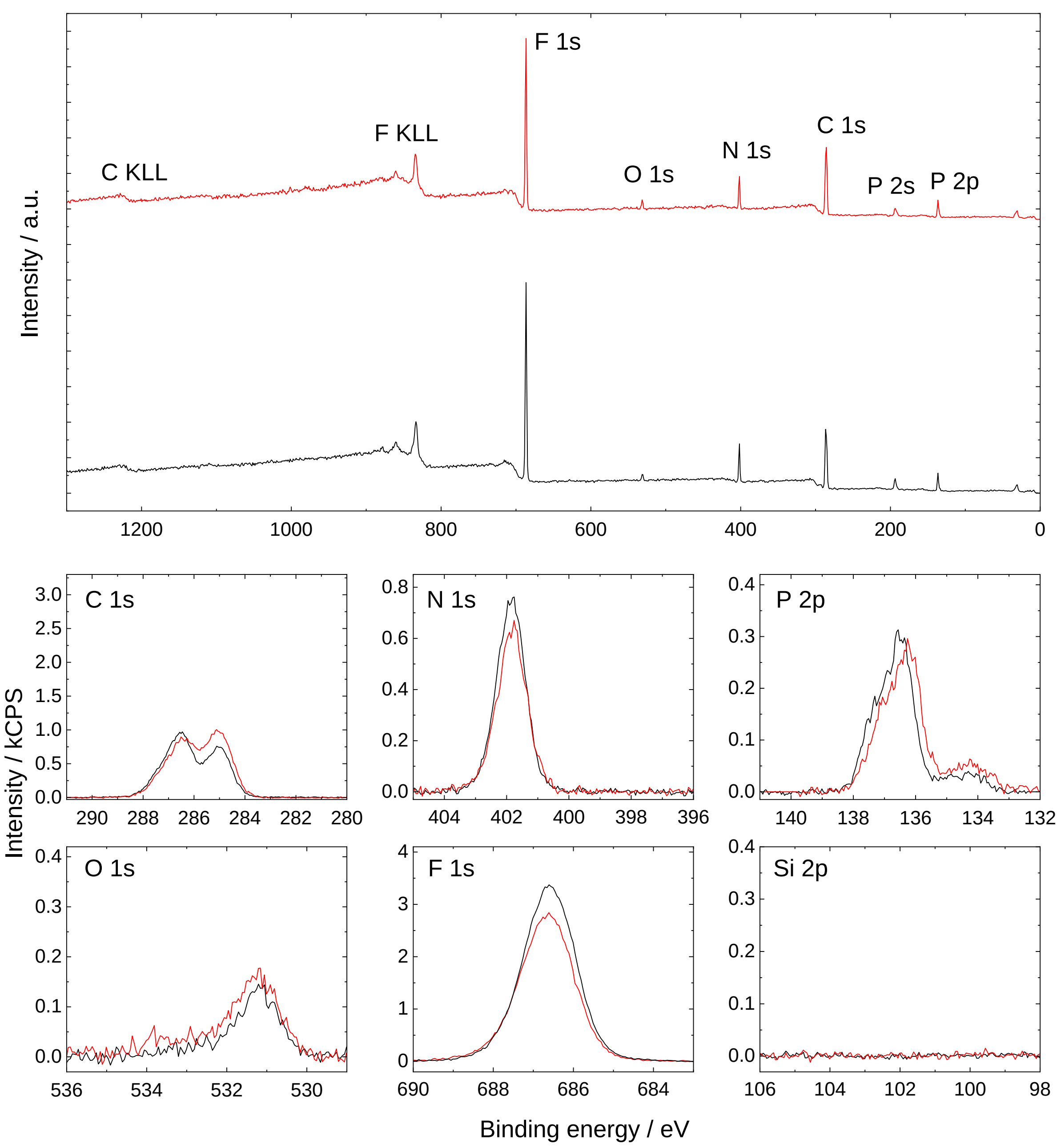

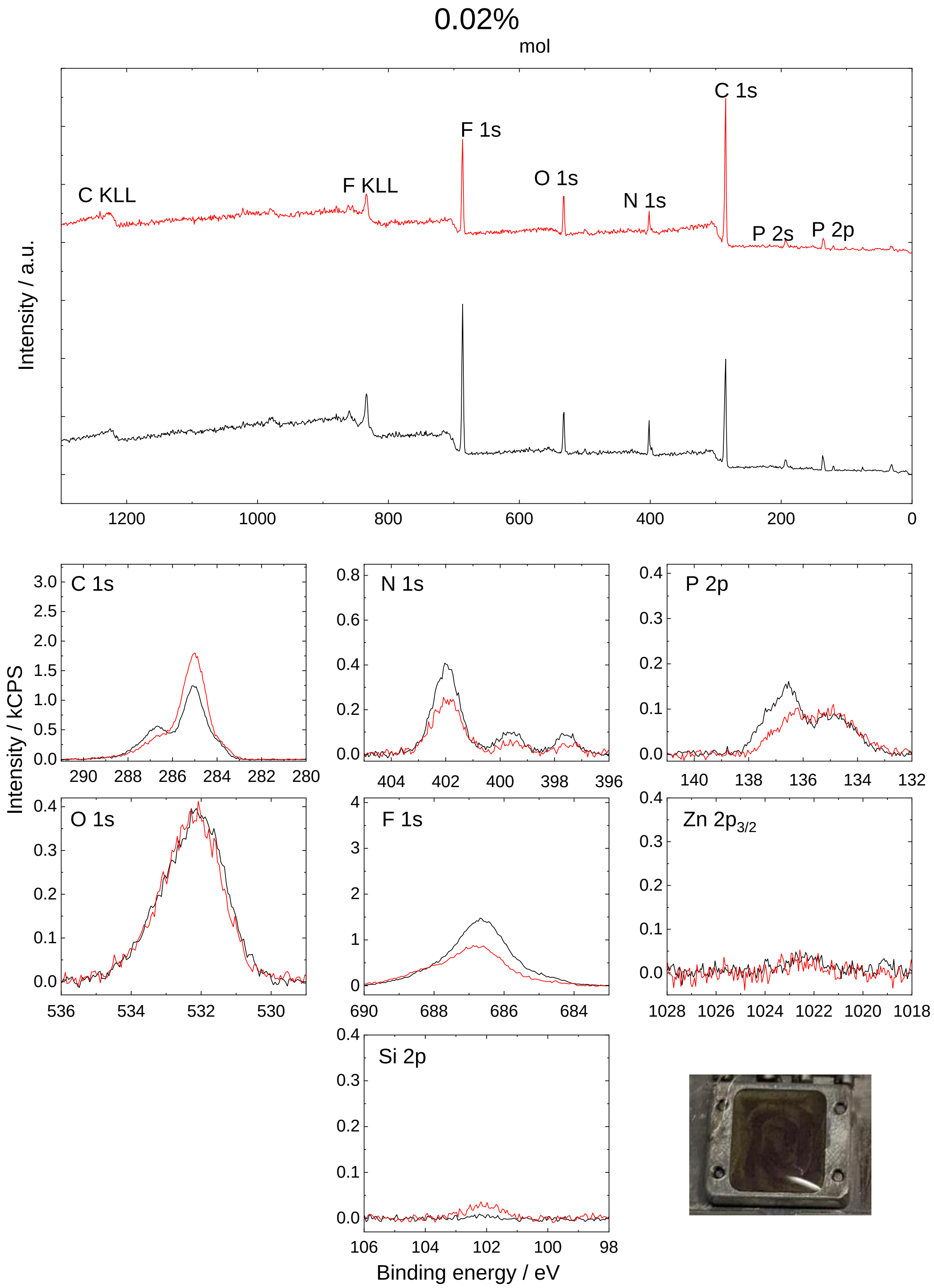

Figure S3: Survey, C 1s, N 1s, Zn $2p_{3/2}$, O 1s, F 1s, , P 2p, and Si 2p XP spectra for 0.02%$_{mol}$ solution of Zn-DDP in $[C_4C_1Im][PF_6]$, at 0° (black) and 80° (red) emission. Photo of the solution in the sample holder.

0.05%$_{mol}$

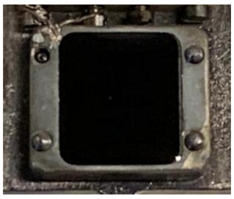

Figure S4: Survey, C 1s, N 1s, Zn 2p$_{3/2}$, O 1s, F 1s, , P 2p, and Si 2p XP spectra for 0.05%$_{mol}$ solution of Zn-DDP in [C_4C_1Im][PF_6], at 0° (black) and 80° (red) emission. Photo of the solution in the sample holder.

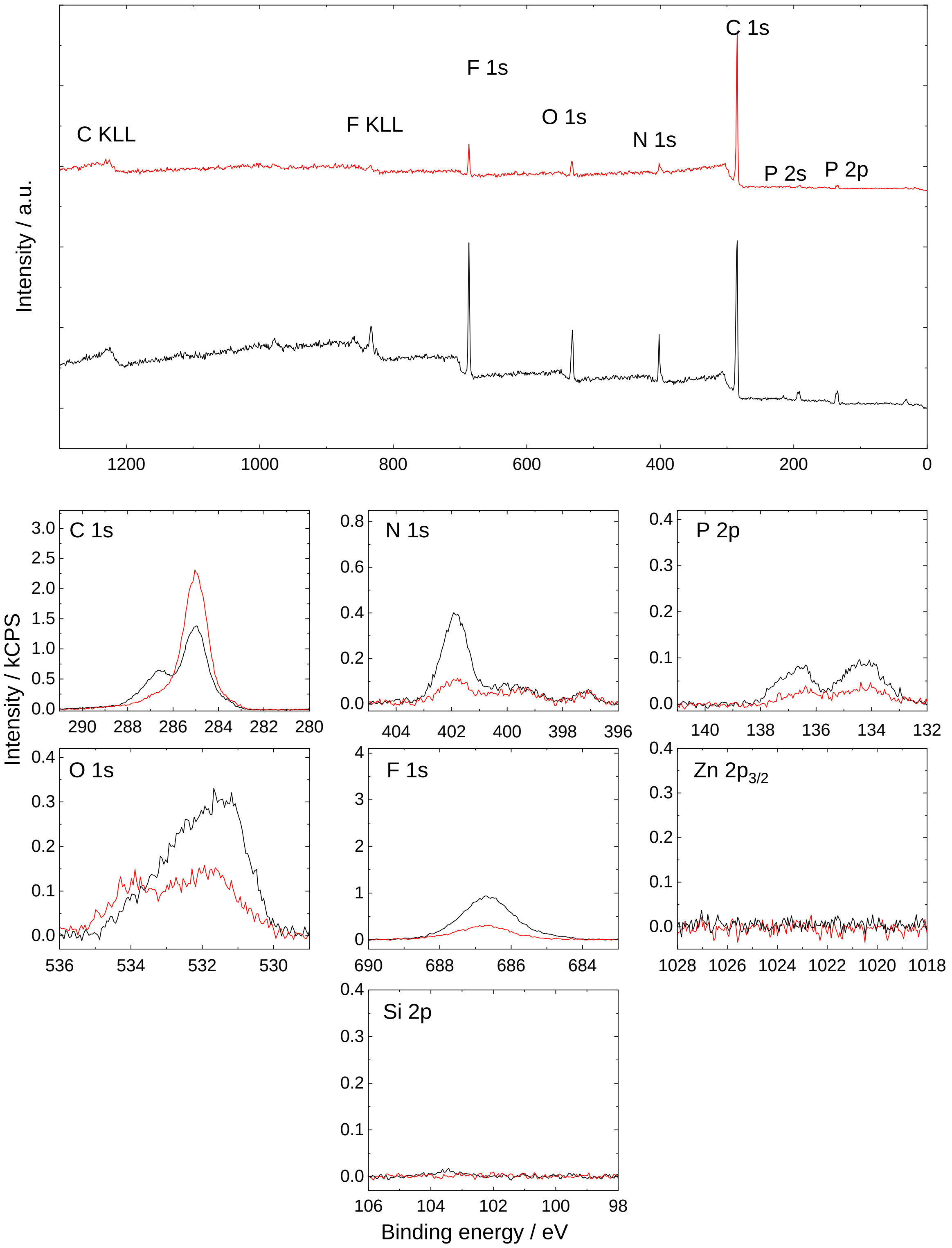
C KLL
F KLL
F 1s
O 1s
N 1s
C 1s
P 2s
P 2p
Intensity / a.u.
1200
1000
800
600
400
200
0
C 1s
N 1s
P 2p
O 1s
F 1s
Zn 2p3/2
Si 2p
Intensity / kCPS
Binding energy / eV

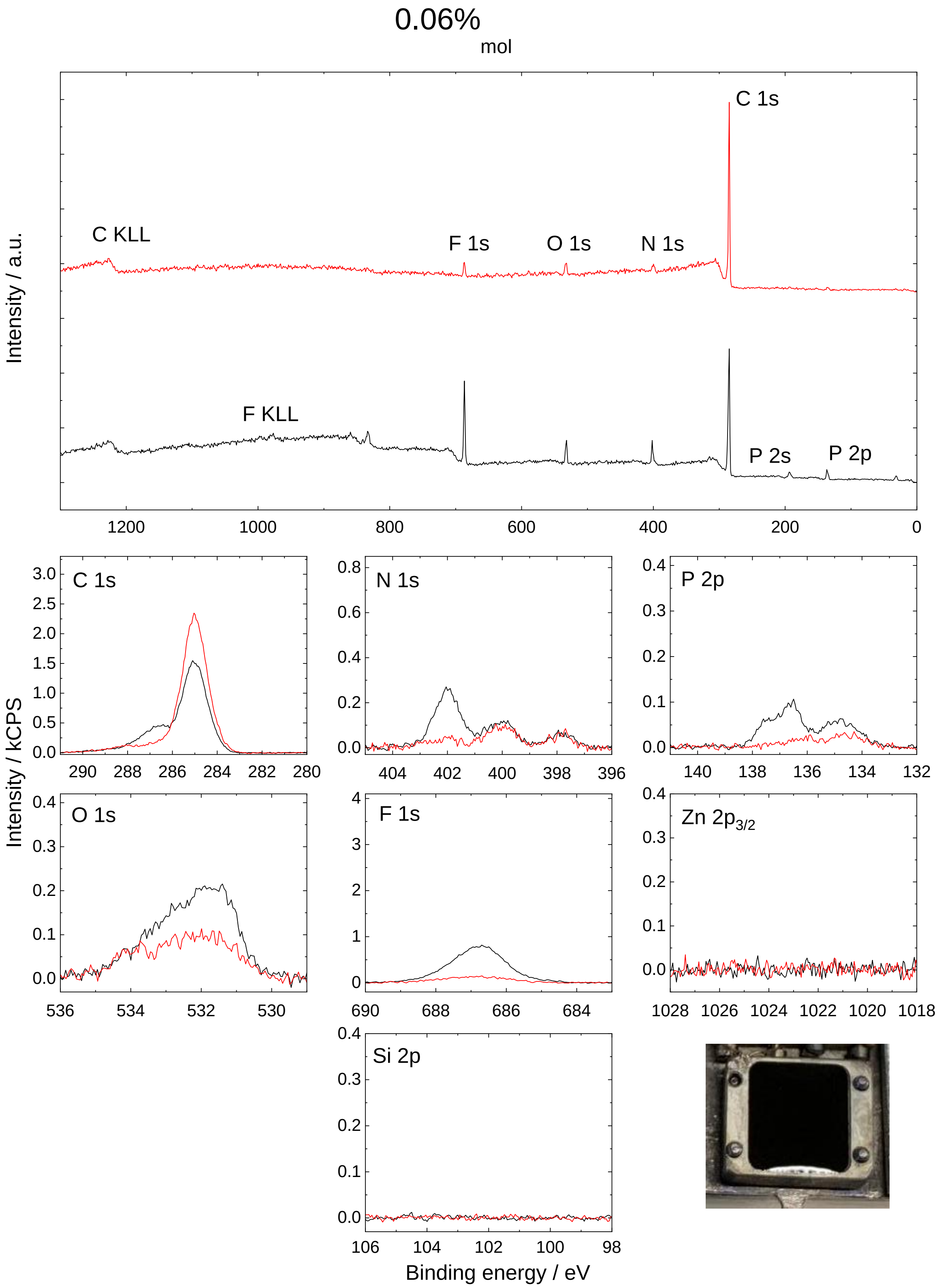

Figure S5: Survey, C 1s, N 1s, Zn $2p_{3/2}$, O 1s, F 1s, , P 2p, and Si 2p XP spectra for $0.06\%_{mol}$ solution of Zn-DDP in $[C_4C_1Im][PF_6]$, at 0° (black) and 80° (red) emission. Photo of the solution in the sample holder.

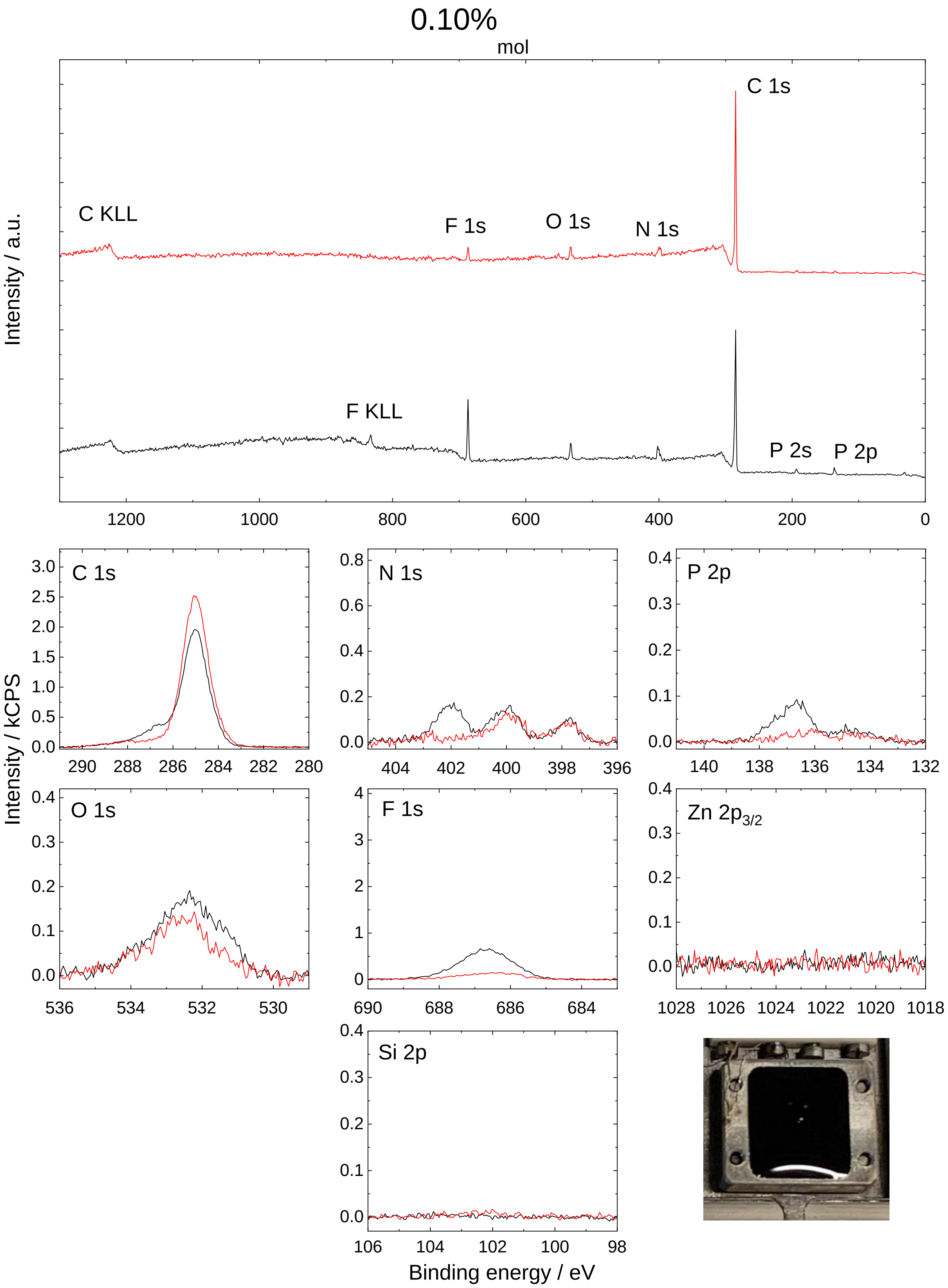

Figure S6: Survey, C 1s, N 1s, Zn $2p_{3/2}$, O 1s, F 1s, , P 2p, and Si 2p XP spectra for $0.10\%_{mol}$ solution of Zn-DDP in $[C_4C_1Im][PF_6]$, at 0° (black) and 80° (red) emission. Photo of the solution in the sample holder.

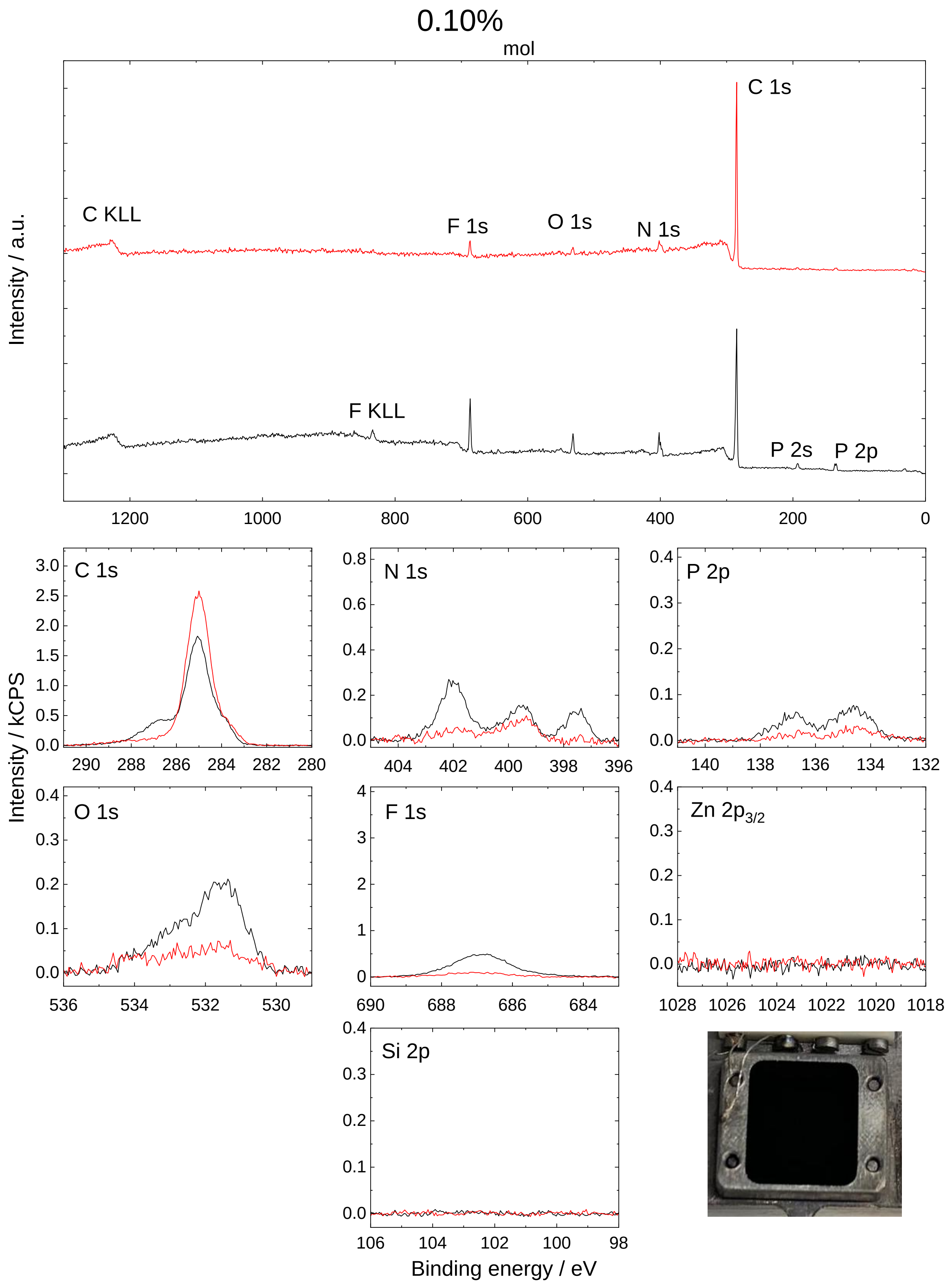

Figure S7: Survey, C 1s, N 1s, Zn $2p_{3/2}$, O 1s, F 1s, , P 2p, and Si 2p XP spectra for 0.10%$_{mol}$ solution of Zn-DDP in $[C_4C_1Im][PF_6]$, at 0° (black) and 80° (red) emission. Photo of the solution in the sample holder.

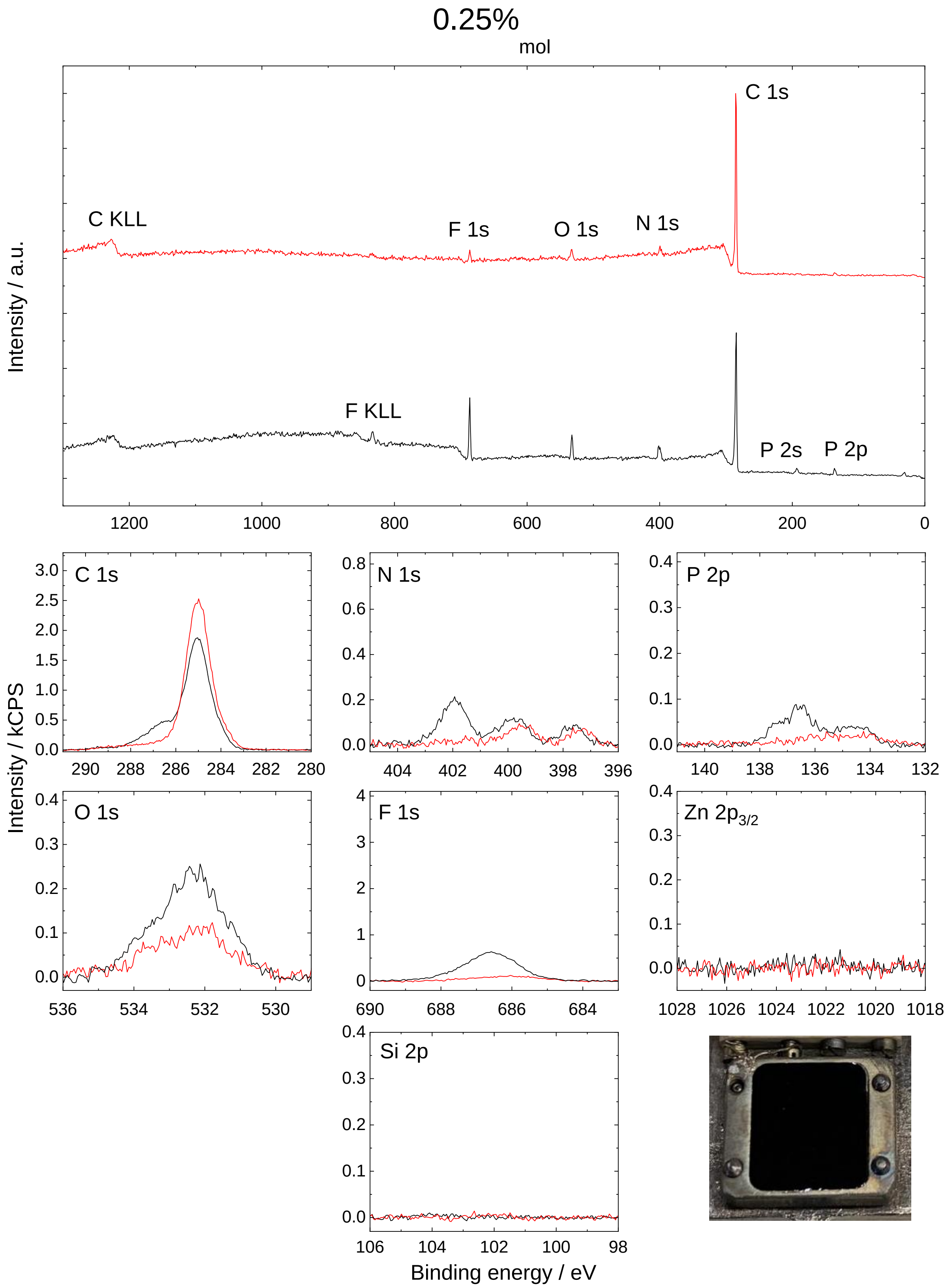

Figure S8: Survey, C 1s, N 1s, Zn $2p_{3/2}$, O 1s, F 1s, P 2p, and Si 2p XP spectra for $0.25\%_{mol}$ solution of Zn-DDP in $[C_4C_1Im][PF_6]$, at 0° (black) and 80° (red) emission. Photo of the solution in the sample holder.

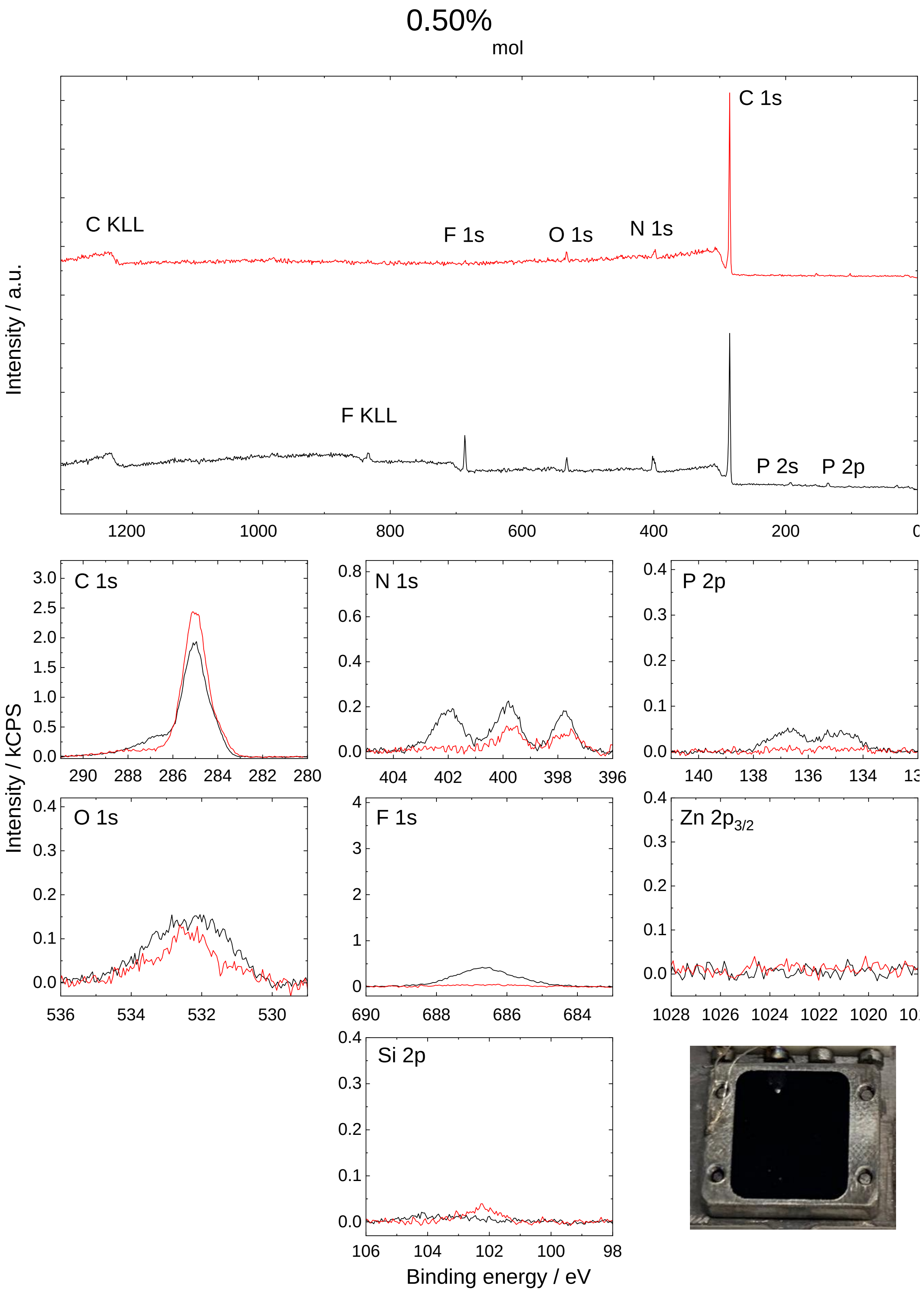

Figure S9: Survey, C 1s, N 1s, Zn $2p_{3/2}$, O 1s, F 1s, P 2p, and Si 2p XP spectra for $0.50\%_{mol}$ solution of Zn-DDP in $[C_4C_1Im][PF_6]$, at 0° (black) and 80° (red) emission. Photo of the solution in the sample holder.

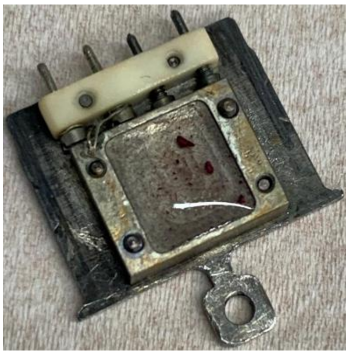

Figure S10: Zn-DDP stirred for ~5h under ambient conditions at RT in $[C_4C_1Im][PF_6]$ shows no sign of dissolution. The photo reveals solid Zn-DPP particles settled at the bottom of the XPS sample holder covered by the clear IL.

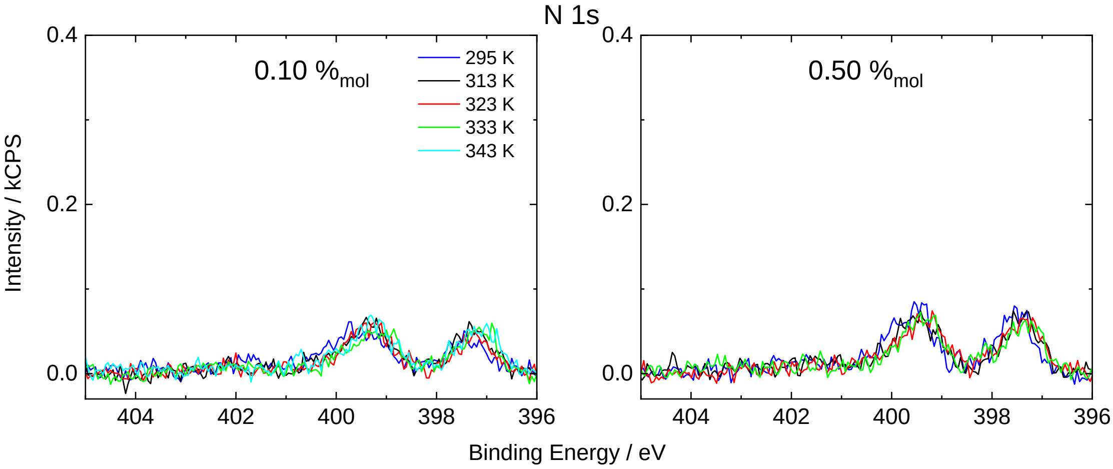

Figure S11: Temperature-dependent XPS measurements for the N 1s region at 80° emission angle for solutions with a Zn-DDP concentration of a) $0.10\%_{mol}$ and b) $0.50\%_{mol}$ in $[C_4C_1Im][PF_6]$

**a) 0.02%$_{mol}$**	C 1s	C 1s	C 1s	N 1s	N 1s	N 1s	F 1s	P $2p_{3/2}$	Zn $2p_{3/2}$

Table S1: Quantitative analysis of the XPS core levels for the solutions of Zn-DDP in [C_4C_1Im][PF_6] with concentrations of a) 0.02%$_{mol}$, b) 0.05%$_{mol}$, c) 0.06%$_{mol}$, d,e) 0.10%$_{mol}$, f) 0.25%$_{mol}$, and g) 0.50%$_{mol}$

	C_2	C_{het}	C_{alk}	N_{Im}	-NH-	=N-			
BE/eV	287.6	286.7	285.0	402.0	399.7	397.6	686.6	136.5	1022.3
Nominal	1.00	4.002	3.01	2.00	0.0004	0.0004	6.00	1.00	0.0002
Exp. 0°	0.59	3.21	7.10	1.38	0.33	0.27	3.66	0.44	0.03
Exp. 80°	0.78	2.13	10.38	0.97	0.18	0.14	2.14	0.27	0.02
**b) 0.05%$_{mol}$**									
BE/eV	287.5	286.6	285.0	401.9	399.8	397.4	686.7	136.5	
Nominal	1.00	4.00	3.02	2.00	0.00	0.00	6.00	1.00	0.00
Exp. 0°	0.76	3.69	8.01	1.44	0.43	0.20	2.25	0.24	0.00
Exp. 80°	0.45	1.54	13.27	0.43	0.32	0.18	0.76	0.09	0.00
**c) 0.06%$_{mol}$**									
BE/eV	287.5	286.6	285.0	402.1	400.1	397.9	686.8	136.6	
Nominal	1.00	4.00	3.02	2.00	0.0011	0.0011	6.00	1.00	0.0006
Exp. 0°	0.92	2.55	9.56	0.95	0.48	0.22	2.07	0.29	0.00
Exp. 80°	0.57	0.95	14.39	0.18	0.37	0.21	0.30	0.05	0.00
**d) 0.10%$_{mol}$**									
BE/eV	287.5	286.6	285.0	402.0	400.0	397.8	686.7	136.6	
Nominal	1.00	4.01	3.04	2.00	0.00	0.00	6.00	1.00	0.00
Exp. 0°	0.72	1.87	11.25	0.66	0.51	0.29	1.50	0.24	0.00
Exp. 80°	0.48	0.67	14.71	0.00	0.45	0.28	0.40	0.07	0.00
**e) 0.10%$_{mol}$**									
BE/eV	287.4	286.5	285.0	402.0	399.6	397.6	686.9	136.6	
Nominal	1.00	4.01	3.04	2.00	0.0021	0.0021	6.00	1.00	0.001
Exp. 0°	1.09	2.25	10.32	0.98	0.54	0.41	1.30	0.16	0.00
Exp. 80°	0.49	0.74	14.61	0.21	0.37	0.31	0.27	0.05	0.00
**f) 0.25%$_{mol}$**									
BE/eV	287.3	286.4	285.0	402.0	399.8	397.7	686.6	136.5	
Nominal	1.00	4.02	3.09	2.00	0.0047	0.0047	6.00	1.00	0.0024
Exp. 0°	1.12	2.34	10.48	0.72	0.43	0.28	1.53	0.23	0.00
Exp. 80°	0.36	0.67	15.09	0.08	0.31	0.22	0.32	0.05	0.00
**g) 0.50%$_{mol}$**									
BE/eV	287.7	286.8	285.0	402.0	399.8	397.8	686.6	136.5	
Nominal	1.00	4.04	3.19	2.00	0.01	0.01	6.00	1.00	0.005
Exp. 0°	0.52	2.09	11.54	0.75	0.66	0.50	1.05	0.14	0.00
Exp. 80°	0.22	0.90	15.22	0.08	0.37	0.30	0.17	0.00	0.00

Proposed surface layer model

For the surface-enriched porphyrin molecules with a buoy-like orientation (that is, the alkyl chains pointing toward the vacuum; see Figure S11), we estimate a thickness, *d*, of ~3 nm, based on the molecular size and C—C bond length within the dodecyl chains. Thus, assuming that for saturated solutions (≥ 0.10$\%_{mol}$) the IL/vacuum interface is composed of a full layer of porphyrins as depicted in Figure S11 with neat IL underneath (note the maximum porphyrin concentration in the bulk is 0.50 $\%_{mol}$), we can use the damping of the N 1s signal of the IL imidazolium cations N_{Im}. Table S2 displays the measured peak N_{Im} areas I_0 for the neat, unheated IL (top row) and I_d for the saturated solutions (rows below). The ratio I_d/I_0 represents the damping of the N_{Im} signal by the porphyrin surface layer. For all solutions, the experimentally obtained value of I_d/I_0 is 0.30 ± 0.02. Considering an exponential damping law $I_d/I_0 = exp(-d/(\lambda \cdot cos\theta))$, a thickness *d* of 3.0 nm, an inelastic mean free path λ of the N 1s electrons of 2.5 nm, and the emission angle θ of 0°, we obtain an I_d/I_0 ratio of 0.30, in excellent agreement with the measured ratios obtained for the saturated solutions in Table S2. This agreement thus supports our assumed model that the porphyrins adopt a buoy-like configuration, forming a full layer of porphyrins at the IL/vacuum interface. One should note, however, that the above consideration ignores the presence of the observed phosphate species, which might also affect the measured N_{Im} intensities, and thus, our model should be considered as a simplified approximation only.

Table S2: Experimental values and ratio for the XPS signal of the N 1s core-level for the N_{Im} species for the neat unheated $[C_4C_1Im][PF_6]$ and the saturated Zn-DDP solutions (≥ 0.10 $\%_{mol}$) recorded at 0° emission angle.

	Peak Area (in 0°)	Ratio (I_d/I_0)
Neat $[C_4C_1Im][PF_6]$	836.4	-
0.10 $\%_{mol}$	237.5	0.28
0.25 $\%_{mol}$	254.4	0.30
0.50 $\%_{mol}$	258.9	0.31

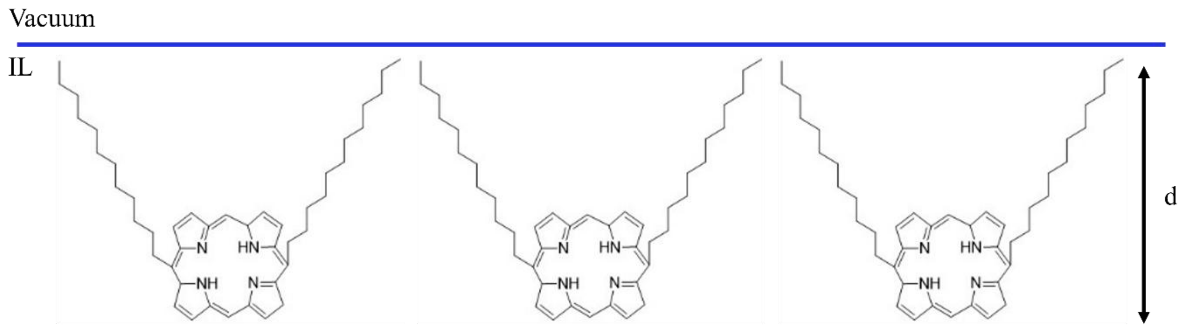

Figure S11: Illustration of the surface-enriched porphyrin molecules at the IL/vacuum interface oriented in a buoy-like configuration forming a closed layer for the saturated solutions. The estimated layer thickness *d* is about 3 nm based on the bond lengths within the molecule.

References

(1) Gottfried, J. M.; Maier, F.; Rossa, J.; Gerhard, D.; Schulz, P. S.; Wasserscheid, P.; Steinrück, H.-P. Surface Studies on the Ionic Liquid 1-Ethyl-3-Methylimidazolium Ethylsulfate Using X-Ray Photoelectron Spectroscopy (XPS). *Z. Phys. Chem.* **2006**, *220*, 1439-1453. DOI: 10.1524/zpch.2006.220.10.1439.
